# Spinal inhibitory interneurons: regulators of coordination during locomotor activity

**DOI:** 10.3389/fncir.2023.1167836

**Published:** 2023-04-20

**Authors:** Simon Gosgnach

**Affiliations:** Department of Physiology, University of Alberta, Edmonton, AB, Canada

**Keywords:** locomotion, interneuron, inhibitory, motor control, neural network

## Abstract

Since the early 1900’s it has been known that a neural network, situated entirely within the spinal cord, is capable of generating the movements required for coordinated locomotion in limbed vertebrates. Due the number of interneurons in the spinal cord, and the extent to which neurons with the same function are intermingled with others that have divergent functions, the components of this neural circuit (now referred to as the locomotor central pattern generator-CPG) have long proven to be difficult to identify. Over the past 20 years a molecular approach has been incorporated to study the locomotor CPG. This approach has resulted in new information regarding the identity of its component interneurons, and their specific role during locomotor activity. In this mini review the role of the inhibitory interneuronal populations that have been shown to be involved in locomotor activity are described, and their specific role in securing left-right, and flexor extensor alternation is outlined. Understanding how these interneuronal populations are activated, modulated, and interact with one another will help us understand how locomotor behavior is produced. In addition, a deeper understanding of the structure and mechanism of function of the locomotor CPG has the potential to assist those developing strategies aimed at enhancing recovery of motor function in spinal cord injured patients.

## Introduction

The act of locomotion, or moving from place to place within one’s environment, is an essential behavior in all non-sessile species. It has long been known that, in mammals, this behavior is controlled by a neural circuit, situated in the spinal cord, referred to as the locomotor central pattern generator (CPG) (Grillner et al., 1998). In limbed vertebrates, the locomotor CPG that is responsible for regulating hindlimb stepping resides in the ventral region of the lumbar spinal cord. In intact mammals descending input from the cortex and brainstem is crucial for locomotor initiation (Leiras et al., 2022), and sensory input is required to modify motor outputs to match the demands of the terrain (Prochazka and Ellaway, 2012). However, studies using the isolated spinal cord have demonstrated that the locomotor CPG, without any additional input, is able to produce intricately coordinated locomotor-like activity in flexor and extensor hindlimb motoneurons on either side of the body (Grillner, 1985; Grillner and Jessell, 2009; Kiehn, 2016). Since a comprehensive understanding of how the locomotor CPG is assembled and operates has the potential to lead to therapeutic approaches to restore movement after spinal cord injury, investigations into the structure and mechanism of function of this neural circuit have been ongoing since its discovery, more than a century ago (Brown, 1911). Since the turn of the century, technological advances in molecular and developmental genetics have resulted in the implementation of a novel experimental approach to study the locomotor CPG. This has led to substantial insight regarding the neuronal components of this neural circuit, and the manner in which they interact with one another (reviewed in Goulding, 2009; Kiehn, 2016). The principal findings of this work is that interneurons in the developing spinal cord can be divided up into 10 “parent” populations (dI1-dI6 and V0-V3), each interneuron within a population being genetically similar to others within the same population, and genetically distinct from those belonging to other populations (Tanabe and Jessell, 1996). Investigation of the migration patterns of each population have indicated that 5 of these reside in the ventral aspect of the lumbar spinal cord postnatally, a location consistent with participation in locomotor activity. Subsequent studies on these populations have incorporated anatomical and electrophysiological techniques to characterize the properties of each. Their specific role during locomotor activity has been investigated by silencing or ablating a given cellular population and identifying locomotor defects that are apparent in their absence (Lanuza et al., 2004; Hinckley et al., 2005; Gosgnach et al., 2006; Crone et al., 2008; Zhang et al., 2008, 2014; Zagoraiou et al., 2009; Andersson et al., 2012; Talpalar et al., 2013; Britz et al., 2015; Haque et al., 2018).

Since the initial identification of these populations, subsequent investigation into their genetic makeup has led to the conclusion that, in most cases, the populations can be further subdivided into multiple subsets based on transcription factor expression downstream of those originally used to define each cell group (Gosgnach et al., 2017). In some cases multiple subpopulations, which are derived from a single “parent” cell group, have complimentary roles during locomotor activity. The integration of these subsets into the working network model of the locomotor CPG has furthered our understanding of this neural circuit by enabling us to better grasp how different groups of muscles are activated sequentially (i.e., muscle synergies recruited) in order to produce the specific locomotor outputs that are required (i.e., those responsible for slow walking vs. fast running- Rybak et al., 2015).

As it currently stands, 9 genetically- defined groups of neurons can be identified in the ventral half of the postnatal spinal cord that have a defined function during locomotor activity, five of these use excitatory neurotransmitter, and 4 populations are inhibitory. The excitatory populations (V0_*V*_, V2a, V3, Shox2, and Hb9 neurons) have a variety of functions such as locomotor initiation (Dougherty et al., 2013), maintenance of locomotor stability (Zhang et al., 2008), and regulation of synchronous activity of motoneurons on either side of the spinal cord (Crone et al., 2008, 2009). In contrast, the inhibitory populations (dI6, V0_*D*_, V1, and V2b groups) each play an essential role in the appropriate coordination of either left-right (Lanuza et al., 2004; Talpalar et al., 2013), or flexor-extensor (Zhang et al., 2014; Britz et al., 2015), alternation. In this mini review I will focus solely on the inhibitory populations, and describe their specific role in coordinating locomotor activity.

## Inhibitory populations involved in left-right alternation: V0 and dI6 populations

Locomotor activity in bipedal mammals consists of alternation between the left and right hind limbs. This is result of a nuanced pattern of activation of various hindlimb muscles, with a great deal of variability in the onsets and offset in each (Engberg and Lundberg, 1969; Rasmussen et al., 1978). Generally speaking, however, when the left limb is on the ground (i.e., in stance phase) the extensor muscles are primarily active and the flexors are primarily silent. In the right limb (which would be in swing phase), the extensor muscles are inhibited while the flexors are active. As the speed of locomotion increases, the amount of time each limb spends in stance phase decreases while the amount of time spent in the swing phase is largely unchanged (Goslow et al., 1973; Halbertsma, 1983). Importantly, at all speeds of locomotion, alternation between the left and right hindlimbs persist. The majority of research into the structure and function of the locomotor CPG has come from quadrupedal species such as the cat or rodent, which have a unique set of muscle synergies as the speed of locomotor activity increases. Slower speed locomotion involves alternation similar to that seen in bipeds, and can be classified as either walk or trot. As speed increases there is synchronous activity across the midline and two similar locomotor gaits, gallop and bound, dominate (Bellardita and Kiehn, 2015). The current theory that accounts for the ability of this neural circuit to generate a variety of stepping patterns holds that the locomotor CPG is a two-layered circuit comprised of distinct population of interneurons. The “top” layer is responsible for rhythm generation, and activating the “lower,” pattern forming layer which, in turn, activates or inhibits motoneurons in a manner appropriate for the required locomotor task (Rybak et al., 2015).

**FIGURE 1:**
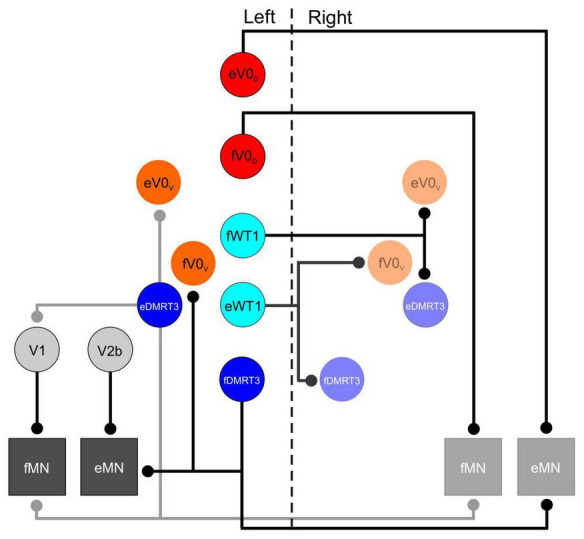
Schematic of synaptic contacts from inhibitory interneuronal populations in the lumbar spinal cord that are involved in locomotion. Vertical dashed line separates left and right side of spinal cord. In this schematic all inhibitory populations that have a defined role during locomotor activity appear on the left side, only those populations that receive inputs from these cells appear on the right side. Motoneurons are shown on both sides. Lowercase f and e indicate those members of a given population that are active during flexion and extension respectively.

The first study to investigate the functional role of one of the molecularly defined interneuronal populations during locomotor activity was focused on the V0 neurons. These cells were shown to originate from progenitors expressing the transcription factor Dbx1, and reside in lamina VIII of the spinal cord postnatally (Pierani et al., 2001). Initially all V0 neurons were analyzed collectively, and this population was considered to be primarily comprised of inhibitory neurons which project commissural axons (Pierani et al., 2001). Subsequent work indicated that the V0 population could be divided into two subsets of neurons which could be distinguished from one another molecularly. The ventral subpopulation (V0_*V*_) can be identified by expression the transcription factor Evx1 as well as Dbx1, and a dorsal population (V0_*D*_) which can be identified by expression of Dbx1 but not Evx1 (Moran-Rivard et al., 2002). In the Dbx1 mutant mouse, in which all V0 neurons are absent, activity on the left and right sides of the spinal cord appeared to be “disconnected” from one another during a locomotor task. Rather that strict alternation between left and right flexor (or left and right extensor) ventral roots, contralateral activity in the Dbx1 mutant mouse drifted in and out, with left and right flexor motor axons sometimes bursting synchronously and sometimes alternating (Lanuza et al., 2004). Interestingly, in this study, mice in which only the Evx1- expressing (V0_*V*_) cells were eliminated failed to show any aberrant left right alternation. This led to the suggestion that the V0_*D*_ subpopulation alone were responsible the coordination of motoneurons on either side of the spinal cord. Subsequent experiments indicating that V0 neurons projected axons toward, and made monosynaptic contacts onto, contralateral motoneurons suggested the circuitry responsible for this function (Lanuza et al., 2004- see Figure 1).

Additional investigation of the differences between the dorsal and ventral subpopulations of V0 neurons revealed a complimentary role for the two subsets during stepping. First, analysis of the neurotransmitter phenotype of each indicated that V0_*D*_ cells were inhibitory while V0_*V*_ cells were excitatory (Pierani et al., 2001; Talpalar et al., 2013). It was also demonstrated that ablation of the V0_*D*_ cells alone resulted in inappropriate left-right alternation at slower locomotor speeds with minimal effect on coordination during faster stepping. Loss of V0_*V*_ cells had the opposite effect, seemingly no impact at slower speeds, but essential for appropriate left/right alternation when locomotor speed increased (Talpalar et al., 2013). The apparent conflict with the previous study (in which removal of V0_*V*_ cell function did not impact left right alternation at all) was likely due to the fact that the speeds generated in the locomotor assay used in the original study did not reach the frequencies at which the V0_*V*_ subpopulation would be recruited, and thus their removal did not affect these slow locomotor outputs. The severity of the locomotor phenotype also differed between the two studies investigating V0 interneuron function during locomotion. In the initial study bilateral activity drifted in and out of phase, indicating the two sides of the spinal cord were “disconnected” from one another (Lanuza et al., 2004) while in the latter study activity on either side of the spinal cord was strictly synchronous (Talpalar et al., 2013). While the differences between the phenotypes has not been directly accounted for, they may have something to do with the mouse models implemented. The latter study used an approach in which diphtheria toxin was produced in select populations, killing cells after they had expressed specific transcription factors (Talpalar et al., 2013). In contrast, the initial study used a mutant mouse model in which V0 cells were not produced and developmental compensation occurred, increasing the number of other neuronal population such as the ipsilaterally projecting V1 cells and the commissural dI6 neurons (Lanuza et al., 2004).

dI6 interneurons have also been shown to play a role in left-right alternation. This population originates from progenitor cells expressing the transcription factors Lbx1 and Dbx1, and is situated immediately dorsal to the V0_*D*_ neurons during embryonic development (Gross et al., 2002; Muller et al., 2002). dI6 neurons can be divided into 2 subsets based on the expression of the postmitotic markers WT1 or DMRT3 (Andersson et al., 2012). Unlike the V0 population, both dI6 subpopulations have been shown to be exclusively inhibitory (Andersson et al., 2012; Haque et al., 2018). A role for dI6 cells in left- right alternation was first suggested in the original study investigating the function of the V0 neurons as it was observed that the number of dI6 neurons increased in the Dbx1 mutant mouse, likely due to developmental compensation (Lanuza et al., 2004). It was suggested that the presence of these dI6 cells, which were known to be inhibitory and commissural, were perhaps responsible for the less severe locomotor phenotypes observed in some of the V0 ablated mice.

A specific role for the DMRT3- expressing neurons in gait coordination was first suggested following the observation that mutation of the DMRT3 gene in horses enabled “pacing” gaits in which the fore and hind limbs on the same side of the body move together while collectively alternating with the fore and hind limbs on the contralateral side of the body (Andersson et al., 2012). Characterization of these cells in mice indicated that, similar to V0_*D*_ neurons, the DMRT3 subset of dI6 cells is primarily situated in lamina VIII of the postnatal spinal cord, and project commissural axons which release the neurotransmitter glycine. Synaptic contacts from this population were observed on motoneurons as well as on premotor neurons in laminae IX (presumably Renshaw cells which belong to the V1 population), as well as cholinergic V0 cells (derived from the V0_*V*_ subpopulation) surrounding the central canal (Andersson et al., 2012- see Figure 1). The aforementioned gait abnormalities observed in horses lacking DMRT3 cells are strongly suggestive of a role for these subset of the dI6 population in left-right alternation. While they have been shown to be rhythmically active during a fictive locomotor task in the mouse (Perry et al., 2019), the locomotor pattern in their absence has yet to be investigated.

The WT1- expressing subset of dI6 neurons share many characteristics with DMRT3 + cells. WT1 neurons are inhibitory, and project commissural axons (Haque et al., 2018). Synaptic boutons from WT1 + cells have been found in close proximity to other commissural interneuronal populations, namely, Evx1- expressing V0_*V*_, as well as DMRT3 interneurons (see Figure 1). Connectivity onto the V0_D_ population was not confirmed, but cannot be ruled out as a postnatal marker for this population was not available (Haque et al., 2018). Like the DMRT3 population, WT1 cells were shown to be rhythmically active during fictive locomotion in the isolated spinal cord preparation (Haque et al., 2018; Schnerwitzki et al., 2018), and silencing of these cells using a DREADD approach resulted in left-right alternation defects (Haque et al., 2018). The locomotor phenotype was less severe than that seen when the V0 neurons were ablated, but clear co-activation of contralateral flexor, and contralateral extensor, motor axons was regularly observed. The conclusion from this study was that the WT1 populations regulated the activity of other commissural interneurons (Haque et al., 2018). Knowing what we know now about the speed dependent regulation of the V0_D_ and V0_V_ interneurons, it would have been interesting to determine whether the WT1 interneurons were also modulated at different locomotor frequencies. For example, one might expect that these cells are actively regulating other commissural populations depending on whether a strictly alternating (walk/trot) or a synchronous gait (bound/gallop) is generated.

## Inhibitory neurons securing flexor-extensor alternation: V1 and V2b populations

While the specific pattern flexor and extensor motoneuron and muscle activation varies during locomotor activity, one general characteristic of locomotor activity in both bipedals, and quadrupedals is the alternation of flexor and extensor motoneurons/muscles. The specific activity profile of flexor and extensor motor pools around the hip, knee, and ankle joints is complex and the peak firing phases of each has been shown to be determined by the output of the locomotor CPG, and modulated by sensory input (Patla, 1985). The specific interneuronal populations that comprise the components of the locomotor CPG which regulate the flexor/extensor alternation around hindlimb joints has only recently been revealed. The hunt for a population of neurons responsible for this alternation has involved silencing or ablating specific interneuronal populations in the hopes of identifying a locomotor phenotype in which inappropriate flexor/extensor activity could be observed, either *in vitro* or *in vivo*.

Surprisingly, ablating each of the genetically-defined interneuronal populations individually revealed no deficits in ipsilateral coordination. Given their characteristics (ipsilaterally projecting axons, and inhibitory neurotransmitter phenotype), the primary candidates to be involved in flexor/extensor alternation were the V1 (Burril et al., 1997; Sauressig et al., 1999) and V2b (Lundfald et al., 2007; Peng et al., 2007) interneurons which are defined by their expression of En1 and Gata3, respectively. Ablation or silencing of the entire V1 population leads to a marked slowing of locomotor activity (Gosgnach et al., 2006) with no change in flexor- extensor alternation when compared to the wildtype animal (Gosgnach et al., 2006; Zhang et al., 2014). From this work it was suggested that V1 INs facilitate the transition between the active and inactive phases of the step cycle (Gosgnach et al., 2006). Inhibiting activity in the V2b population alone, by arresting synaptic transmission in these cells, has only a very minor effect on left right alternation (Zhang et al., 2014). Although ablation of the function of either of these populations in isolation had little effect on ipsilateral alternation, experiments in which both of these populations were ablated simultaneously, resulted in the complete collapse of flexor- extensor alternation in the isolated spinal cord, resulting in co-activation of flexor and extensor motor axons on the same side of the body, a locomotor phenotype that is not otherwise observed in either bipeds or quadrupeds, suggesting that the V1 and V2b neurons work together to maintain flexor-extensor alternation.

Due to expression of the markers V1 (En1) and V2b populations (Gata3) throughout the CNS, inhibiting the function of these cells in the aforementioned study was lethal at either late embryonic, or early postnatal time points. This dictated that the experiments used to identify the V1 and V2b cells are required for ipsilateral alternation were carried out on isolated spinal cords removed from immature mice, which limited the understanding of how they carried out this function. A subsequent study by the same group used a tripartite genetic approach to insert a foreign diphtheria toxin receptor solely into those neurons which expressed En1 and/or Gata3, and were located in the spinal cord (Britz et al., 2015). Application of the ligand for this diphtheria toxin receptor ablated these neurons and enabled their function during locomotor activity to be examined in greater detail. Furthermore, the use of postnatal animals also allowed the synaptic connectivity of these neurons to be analyzed allowing the specific role of each to be elucidated. Using this approach it was demonstrated that mice lacking V1 cell function displayed limb hyperflexion, while the limbs of those mice lacking V2b neuronal function had their hindlimbs locked in hyperextension (Britz et al., 2015). These findings nicely complimented anatomical data which indicated that V1 neurons preferentially contacted flexor motoneurons while the V2b population tended to terminate on extensor motor pools (Britz et al., 2015- see Figure 1). Given the inhibitory nature of both populations the data suggests that the loss of function of the V1 cells results in lack of inhibition of flexor motor neurons (and thus hyperflexion) while loss of V2b function results in insufficient inhibition of extensor motoneurons (and excessive limb extension). A balance of the two cell populations is required in order to establish coordinated activity of flexors and extensors during locomotion.

## Conclusion

The locomotor CPG had long been considered to be a “black box,” a mysterious neural network that was capable of generating a wide variety of motor synergies when activated. Since the turn of the century incorporation of a multidisciplinary approach involving molecular genetics, anatomy, and electrophysiology has allowed tremendous strides forward to be taken in our ability to identify neurons that comprise this neural network, and also identify their specific function during stepping. The inhibitory neurons that have been identified to be part of this locomotor CPG have thus far been proven to be essential components required for coordination of antagonist motor pools ipsilaterally, or agonist motor pools on either side of the spinal cord in limbed vertebrates. These inhibitory populations are essential for the generation of coordinated movements. As we learn more about the axonal projection pattern of these populations, and the upstream sites that contact these inhibitory populations it is likely to help us understand the processes by which they are modulated in order to alter the activation pattern of muscles that is required as the frequency of locomotor activities changes. Ultimately, a detailed characterization of these, and other interneurons that are components of the locomotor CPG, and a better understanding of how they regulate motoneuron activity, will help us understand how motor behavior is produced, and will provide key information to those developing therapies aimed at enhancing functional recovery of motor control after damage to the CNS.

## Author contributions

The author confirms being the sole contributor of this work and has approved it for publication.
